# The *Leishmania donovani* SENP Protease Is Required for SUMO Processing but Not for Viability

**DOI:** 10.3390/genes11101198

**Published:** 2020-10-14

**Authors:** Annika Bea, Constanze Kröber-Boncardo, Manpreet Sandhu, Christine Brinker, Joachim Clos

**Affiliations:** 1 Leishmaniasis Group, Bernhard Nocht Institute for Tropical Medicine, D-20359 Hamburg, Germany; annika.bea@bnitm.de (A.B.); kroeber@bnitm.de (C.K.-B.); manpreet.sandhu9898@gmail.com (M.S.); brinker@bnitm.de (C.B.); 2 Boehringer Ingelheim RCV, A-1121 Vienna, Austria

**Keywords:** *Leishmania*, SENP, Ulp2, SUMO, CRISPR, protease

## Abstract

The protozoan parasite *Leishmania donovani* is part of an early eukaryotic branch and depends on post-transcriptional mechanisms for gene expression regulation. This includes post-transcriptional protein modifications, such as protein phosphorylation. The presence of genes for protein SUMOylation, i.e., the covalent attachment of small ubiquitin-like modifier (SUMO) polypeptides, in the *Leishmania* genomes prompted us to investigate the importance of the sentrin-specific protease (SENP) and its putative client, SUMO, for the vitality and infectivity of *Leishmania donovani*. While SENP null mutants are viable with reduced vitality, viable SUMO null mutant lines could not be obtained. SUMO C-terminal processing is disrupted in SENP null mutants, preventing SUMO from covalent attachment to proteins and nuclear translocation. Infectivity *in vitro* is not affected by the loss of SENP-dependent SUMO processing. We conclude that SENP is required for SUMO processing, but that functions of unprocessed SUMO are critical for *Leishmania* viability.

## 1. Introduction

*Leishmania donovani* is a protozoan parasite that causes the lethal visceral leishmaniasis, also known as *Kala azar*. It is a vector-borne pathogen, transmitted by female sandflies of the genus *Phlebotomus*, in particular *P. argentipes*. *Leishmania* exists in two main developmental stages. Promastigotes, elongated flagellates, proliferate rapidly in the sandfly gut. When transmitted to humans, the parasites are phagocytized by antigen-presenting cells and once inside the phagosomes, convert into ovoid, aflagellated amastigotes as which they may persist in the host for months or years.

The leishmaniae differ from their human host and from most other eukaryotes by their lack of gene-specific transcription regulation [1,2,3], relying on modulated RNA stability [4], inducible translation [5] and reversible gene amplification [6,7] instead.

In addition, *Leishmania* spp. have a full complement of protein kinases [8] and phosphatases [9] to modulate protein activity via phosphorylation and dephosphorylation. Heat shock proteins are important substrates for life cycle stage-dependent phosphorylation [10], but protein kinases also affect parasite morphology, infectivity and viability [8,11,12,13]. Methylation, acetylation and glycosylation of proteins, i.e., modifications of amino acid side chains, have also been described for *Leishmania* [14,15].

Another type of post-translational protein modifications (PTMs), the conjugation of modifying polypeptides to target proteins is not as well researched in *Leishmania*, but known to exist, e.g., the conjugation of a mitochondrial associated ubiquitin fold modifier (UFM) [16,17]. Conjugation of another modifier, small ubiquitin-like modifier (SUMO) was studied in *Trypanosoma* spp: SUMOylation of proteins was described for *Trypanosoma cruzi* [18] and *T. brucei* [19,20], where this PTM is involved in surface antigen expression and nuclear organization. A putative ortholog of *SUMO* is present in the *L. donovani* genome and expressed [5,21].

For SUMOylation to happen, the SUMO precursor must first undergo a proteolytic cleavage by a sentrin-specific protease (SENP), which removes the C-terminal amino acids and leaves an exposed, reactive, C-terminal di-glycine group [22,23]. A putative SENP ortholog is also encoded in the *L. donovani* genome and expressed [5,24]. In humans, the di-glycine is further activated by the E1 protein, transesterified to the E2 SUMO-conjugating enzyme and finally transferred to the substrate protein by the E3 SUMO ligase. DeSUMOylation is also facilitated by SENP [25], establishing SENP as a pivotal enzyme to control the SUMOylation state of substrate proteins.

SUMOylation of proteins may have different consequences and result in (i) interference with binding of partner proteins, (ii) additional interaction sites for other proteins, or (iii) SUMO-induced conformational changes of the modified protein [23]. SUMOylation may interfere or promote other PTMs, such as phosphorylation [26] or ubiquitination [27,28]. The SUMOylation status of proteins is highly dynamic, dependent on cell cycle phases, differentiation and stress exposure [23]. Incorrect or excessive SUMOylation is also associated with severe disease, such as cardiovascular or neurological dysfunctions, but also cancer [28]. It is therefore conceivable that in an organism such as *Leishmania*, which is highly dependent on post-transcriptional gene expression regulation, SUMOylation of proteins may play an important role in its adaption to vectors and hosts.

Here, we describe a reverse genetic analysis of SUMO and SENP in *L. donovani*. We test the SUMO-specific proteolytic activity of SENP *in vivo* and examine its impact on vitality and intracellular survival.

## 2. Materials and Methods

### 2.1. Leishmania Culture Conditions

*Leishmania donovani* strain 1S [29] promastigotes and derived mutants were cultured at 25 °C in M199+ medium [30] with the respective antibiotics: puromycin (25 µg/mL, AppliChem, Darmstadt, Germany), blasticidin (5 µg/mL), G418 (50 µg/mL) and hygromycin B (50 µg/mL, all Carl Roth, Karlsruhe, Germany). Cells were passaged every 3–4 days.

### 2.2. Electrotransfection of Leishmania Parasites

Electrotransfection and selection was performed as described [31]. Clonal parasite populations were obtained by limiting dilution in 96-well plates with an initial inoculum of 0.5 parasites/well in a final volume of 200 µL M199+ medium supplemented with the respective antibiotics and 1× penicillin/streptomycin (Sigma Aldrich, Munich, Germany).

### 2.3. In Vitro Infection of Murine Bone Marrow-Derived Macrophages

Isolation and *in vitro* infection of murine bone marrow derived macrophages was performed as described [30].

### 2.4. Construction and Preparation of Recombinant DNA

The SUMO (LdBPK_080480) and SENP (LdBPK_262070) coding sequences were amplified from *L. donovani* 1S genomic DNA using specific primer pairs (Table S1) that introduce restriction sites as indicated. PCR products were subsequently ligated into pCL2N [32], or derived plasmids pCL2N-3×HA (N-ter) and pCL2N-3×HA (C-ter), predigested with the cognate restriction enzymes. Plasmids were amplified in *Escherichia coli* DH5α and purified by CsCl density gradient ultracentrifugation as described previously [33].

### 2.5. PCR-Amplification of Targeting Constructs

For CRISPR/Cas9-mediated gene disruption, sgRNA templates and replacement constructs were PCR-amplified using the Expand High Fidelity PCR System (Roche, Mannheim, Germany) and PCR conditions essentially as described previously [34]. Oligonucleotides used are listed in Table S1.

### 2.6. RNA Extraction, cDNA Synthesis and Quantitative Real-Time PCR (qRT-PCR)

RNA extraction, cDNA detection and RT-qPCR were performed essentially as described [35,36]. Primer sequences are listed in Table S2.

### 2.7. Next Generation Sequencing

Isolation of genomic DNA, DNA library preparation and sequencing was performed following established protocols and carried out on a MiSeq sequencer (Illumina, San Diego, CA, USA) [36].

### 2.8. Western Blotting

Western blot was performed essentially as described [37,38]. Primary anti-HA IgG antibody (polyclonal, mouse, 1:5000; Invitrogen, Carlsbad, CA, USA) in blocking solution (5% milk/TBST solution) was used in conjunction with anti-mouse-AP IgG (polyclonal, goat, 1:1000; Dianova, Hamburg, Germany).

### 2.9. Immunofluorescence Assays

Immunofluorescence assays of log-phase promastigotes, heat-shocked promastigotes and axenic amastigotes were performed as described previously [38]. Briefly, 2 × 10^5^ cells were washed with 1×PBS and applied on microscopic slides and fixed with ice-cold methanol. Following permeabilization and blocking, the cells were stained with primary anti-HA IgG antibody (polyclonal, mouse, 1:3000; Invitrogen, Carlsbad, CA, USA) and secondary anti-mouse Alexa Fluor^®^ 594 IgG (polyclonal, goat, 1:1000; Thermo Fisher Scientific, Waltham, MA, USA) and DAPI (1:50; Sigma Aldrich, Munich, Germany). Fluorescence microscopy was carried out on an EVOS^®^ FL Auto Cell Imaging System using a 64× magnification.

### 2.10. In Silico Procedures

In silico construction of plasmids, DNA and protein sequence analyses was performed using the MacVector software, version 17 (MacVector Inc., Cambridge, UK). Microscopy images were processed using Adobe Photoshop CS3 (Adobe Corp., San Jose, CA, USA) and juxtaposed using Intaglio (Version 3.9, Purgatory Design, Durango, CO, USA). Multi-panel figures were also assembled using the Intaglio software.

In silico design of *SUMO*- and *SENP*-specific sgRNAs and primers for the amplification of the donor repair cassettes was performed using the LeishGEdit online tool [39]. Oligonucleotides were purchased from Sigma-Aldrich (München, Germany).

Gene annotations and reference genomes (version 42) of *L. donovani* BPK were downloaded from the TriTrypDB server. Reads were aligned to the reference genomes using the MacVector software version 17 and Bowtie2 algorithm [40].

Statistical analyses were performed using Prism (version 8, GraphPad Software, San Diego, CA, USA). Ranking tests were performed using the U-test [41]. Differences were considered significant at a level of *p* < 0.05.

## 3. Results

### 3.1. Expression of SUMO and SENP Proteins in Leishmania spp.

**Figure 1:**
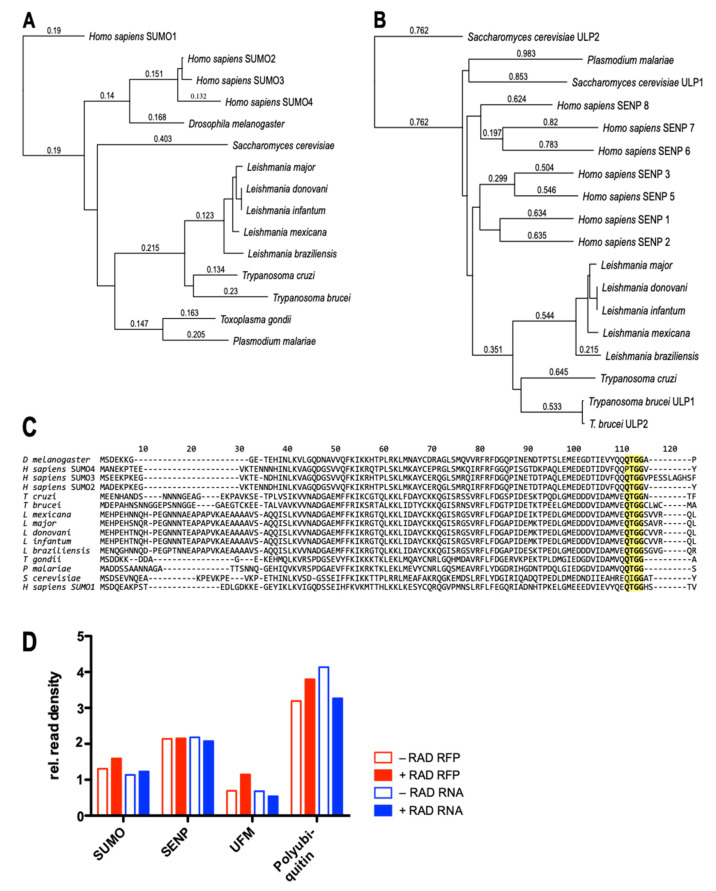
Conservation and expression of *SUMO* and *SENP* in *Leishmania*. (**A**) Phylogenetic analysis of SUMO proteins. Sequence alignment and tree building were done using the neighbor joining algorithm and best-fit analysis with Poisson correction. Numbers indicate amino acid sequence deviation. (**B**) Phylogenetic analysis of SENP proteins, performed as in (**A**). (**C**) Alignment of SUMO amino acid sequences, with the C-terminal di-glycine highlighted. (**D**) Gene expression analysis by ribosome profiling and RNA-seq analysis for *L. donovani* before (−RAD) and after (+RAD) radicicol-induced differentiation. Shown are relative read densities, normalized to the median read densities, for protein synthesis (RFP) and RNA abundance (RNA). Data collected from [5].

When screening the *L. donovani* genome database using BLAST, we identified genes coding for SUMO (LdBPK_080480) and SENP (LdBPK_262070). A ClustalW amino acid sequence comparison of SUMO genes from five *Leishmania* species and two *Trypanosoma* species with four human paralogs and orthologs from *Drosophila* and yeast was performed and used to build a phylogenetic tree (Figure 1A). The SUMO orthologs from the lower eukaryotic clade are distinct from the metazoan SUMOs, but reasonably well conserved (Figure 1C). Notably, the di-glycine motif near the C terminus is present in all SUMO orthologs. The SENP/Ulp2 peptidases, too, were highly conserved among the *Leishmania* spp. and clearly related to the *Trypanosoma* orthologs (Figure 1B).

Both SUMO and SENP are constitutively expressed in *L. donovani*. RNA-seq and ribosome profiling data generated previously [5] show minor variations for SUMO protein synthesis and RNA abundance for *L. donovani* before and after radicicol-induced promastigote-to-amastigote differentiation (Figure 1D). SENP also shows a constitutive, stage-independent protein synthesis and RNA levels. The normalized [5] ribosome footprinting read densities for SUMO and SENP were slightly above those for ubiquitin fold modifier (UFM, LdBPK_161100), another PTM polypeptide [19,20], and lower than those recorded for polyubiquitin (LdBPK_090950), indicating a gene expression rate slightly above the median (1.0) for *L. donovani* genes. With expression of SUMO and SENP established, we decided to target both genes for replacement, using a CRISPR/Cas9 approach.

### 3.2. Replacement of L. donovani SUMO

**Figure 2:**
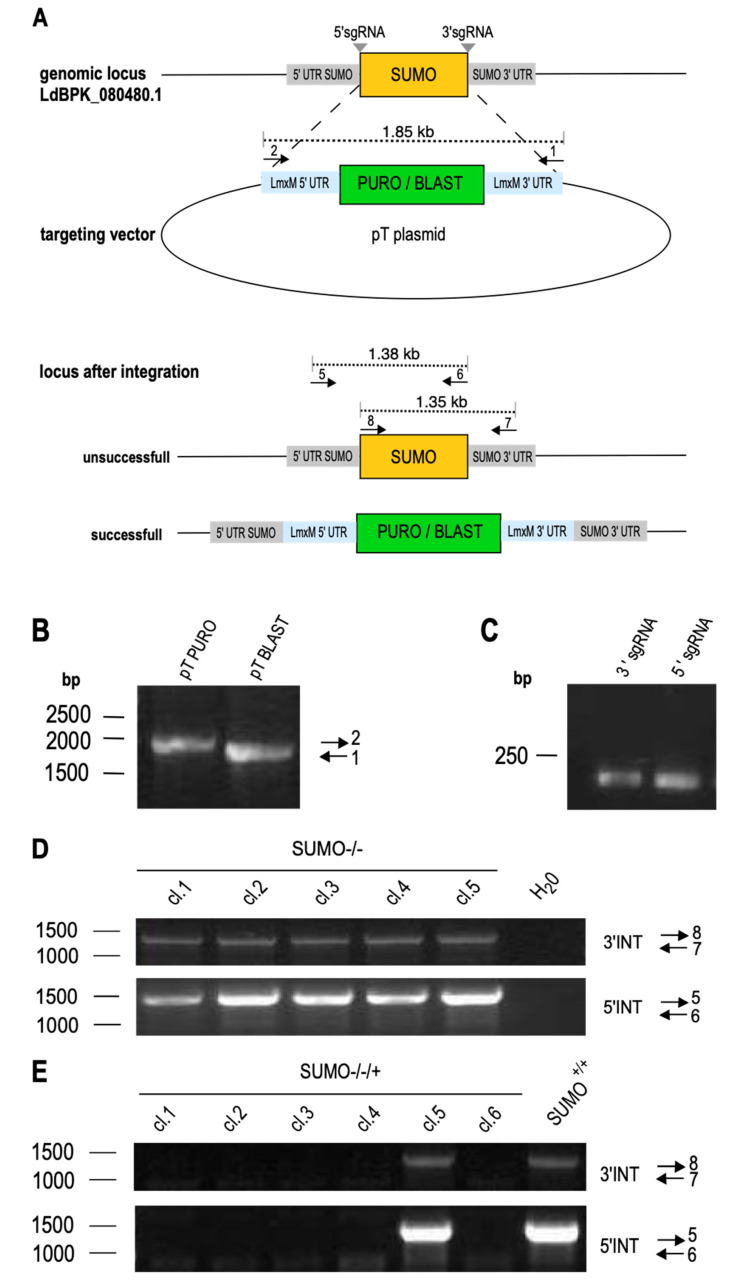
Replacement of *SUMO* in *Leishmania donovani.* (**A**) Schematic representation of LdBPK_080480.1 replacement using the CRISPR/Cas9 technology. *SUMO*-targeting sgRNAs (grey) and the replacement cassettes were PCR-amplified and transfected into a Cas9/T7-RNAP-expressing *L. donovani* strain. Two sets of genotyping primers were used to test for the presence of the gene of interest (GOI) (**B**) Gene-specific replacement cassettes amplified from pTPURO or pTBLAST vector were analyzed by agarose gel electrophoresis and ethidium bromide staining. The position of the DNA size marker is indicated on the left, the primers used are indicated on the right. (**C**) Amplified sgRNA-coding sequences were separated on a 1% agarose gel and stained with ethidium bromide. (**D**) Genotyping of putative gene replacement mutant clones with primer pairs 7+8 or 5+6. PCR products were analyzed by 1% agarose gel electrophoresis. Positions of DNA size markers are shown to the left; the primer pairs are indicated on the right. (**E**) Genotyping of gene replacement mutants in the SUMO over expression background (SUMO^−/−/+^) indicated primer pairs.

To test the importance of *SUMO* for *L. donovani* viability and/or proliferation, we targeted *SUMO* for CRISPR/Cas9-mediated replacement, following an established protocol [34,39]. The 5′sgRNA- and 3′sgRNA-coding sequences, along with the upstream and downstream flanking primers were designed as shown in Figure 2A, together with two primer pairs to test for the presence of *SUMO*. The selection marker gene cassettes from plasmids pTPURO and pTBLAST were amplified using the upstream and downstream flanking primers to yield 1.9 kb PCR products (Figure 2B). Those, together with the 5′- and 3′-sgRNA-coding oligonucleotides (Figure 2C) were transfected into *L. donovani* expressing both the Cas9 recombinase and the T7 RNA polymerase (*L. donovani* (Cas9/T7-RNAP)). The transfectants were then selected under IC_95_ (95%-inhibiting concentration) for puromycin and blasticidin. Selected parasites were cloned by limiting dilution [42] and tested for the presence of *SUMO* by PCR with two independent primer pairs. Figure 2D shows that all tested clones remained positive for *SUMO*.

The success of CRISPR-mediated gene replacement is very dependent on a perfect match between gene sequences and the annealing sgRNA regions. We therefore tested whether the sgRNA pair was able to basepair with the *SUMO* coding sequence. For this, we repeated the transfection of sgRNA-coding oligonucleotides and selection marker cassettes in an *L. donovani* strain over expressing *SUMO* from episomal gene copies to create SUMO^–/–/+^ parasites. In five out of six clones, we could verify the loss of the chromosomal *SUMO* alleles. This confirms the specificity of the sgRNAs and selection marker cassette amplificates. We conclude that replacement of *SUMO* is only possible in the presence of ectopic SUMO gene copies, giving strong evidence for an essential role of *SUMO* in viability and/or proliferative capacity of *L. donovani*.

As C-terminal processing by SENP/Ulp2 is thought to be critical for conjugation and polymerization of SUMO, but also for de-SUMOylation, we next targeted the putative SENP ortholog for replacement.

### 3.3. Replacement of SENP

**Figure 3:**
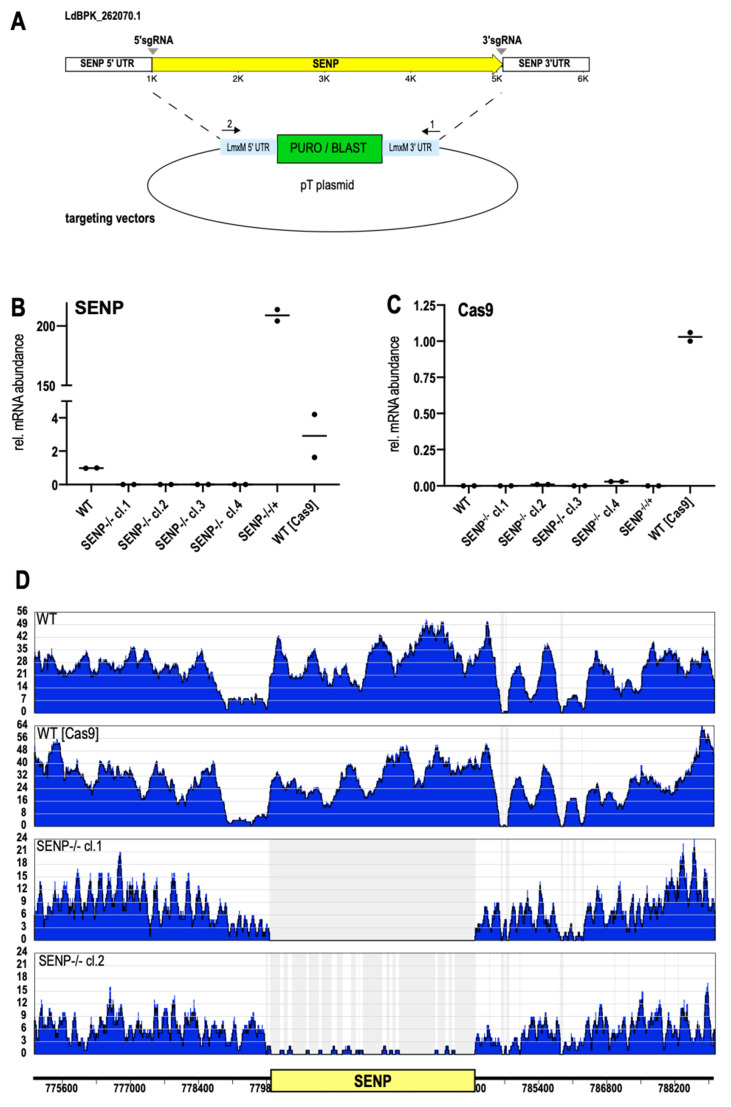
Replacement of SENP. (**A**) Schematic representation of LdBPK_262070 replacement using the CRISPR/Cas9 technology. *SUMO*-targeting sgRNAs (grey arrowheads) and replacement cassettes were PCR-amplified and transfected into *L. donovani* (Cas9/T7RNAP). (**B**,**C**) RT-qPCR of RNA from *L. donovani* wild type (WT), SENP^−/−^ clones 1–4, SENP^−/−^ cl.1[pCLN-SENP], and *L. donovani* (Cas9/T7RNAP). (**B**) SENP-specific RT-qPCR. (**C**) Cas9-specific RT-qPCR. *n* = 2. (**D**) Whole genome sequencing of *L. donovani* wild type (WT), *L. donovani* (Cas9/T7RNAP), SENP^−/−^ cl.1 and SENP^−/−^ cl.2. Sequence reads were aligned to *L. donovani* chromosome 26. The ruler shows the position of the SENP CDS; the numbers refer to the position within chromosome 26. Read coverage is shown in blue.

Again, we used the LeishGedit toolbox to design 5′- and 3′-sgRNAs. Selection marker cassettes were amplified from the pTPURO and pTBLAST plasmids with ends targeting the SENP UTR sequences (Figure 3A). A mix of amplified sgRNA coding DNA and amplified selection marker cassettes was then transfected into *L. donovani* (Cas9/T7-RNAP). The transfectants were then cultivated under puromycin/blasticidin double selection. Selected parasite populations were then subjected to limiting dilution to raise putative SENP^−/−^ clones. RT-qPCR analysis of SENP RNA confirmed the lack of GOI-specific RNA for all selected clones, confirming them as null mutants (Figure 3B). Reintroduction of SENP as an episomal gene copy into clone#1 resulted in a massive over production of SENP RNA (SENP^−/−/+^, Figure 3B). Given the confounding potential of Cas9 expression in the mutants, we analyzed them for Cas9 RNA as well (Figure 3C). Only *L. donovani* (Cas9/T7-RNAP) kept under the episome-specific antibiotic selection showed detectable levels of Cas9 RNA while the SENP^−/−^ mutants had lost the expression plasmid during selection and cloning.

To confirm the loss of SENP on a genomic level, we also performed whole genome sequencing of genomic DNA (gDNA) from *L. donovani* wild type, *L. donovani* (Cas9/T7-RNAP), *L. donovani* SENP^−/−^ cl.1 and *L. donovani* SENP^−/−^ cl.2. Next generation sequencing reads were then aligned to *L. donovani* chromosome 26, using the Bowtie2 algorithm. As expected, both wild type and the Cas9/T7 strain showed uninterrupted read coverage over the SENP gene locus. Conversely, the SENP^−/−^ cl.1 showed a complete lack of SENP-specific reads, while clone 2 showed minimal read coverage, possibly indicating a mosaic population (Figure 3D). However, RT-qPCR analysis (Figure 3B) did not show a low level SENP RNA presence. Still, we chose clone 1 for our further analyses.

### 3.4. SENP Processes the SUMO C Terminus

**Figure 4:**
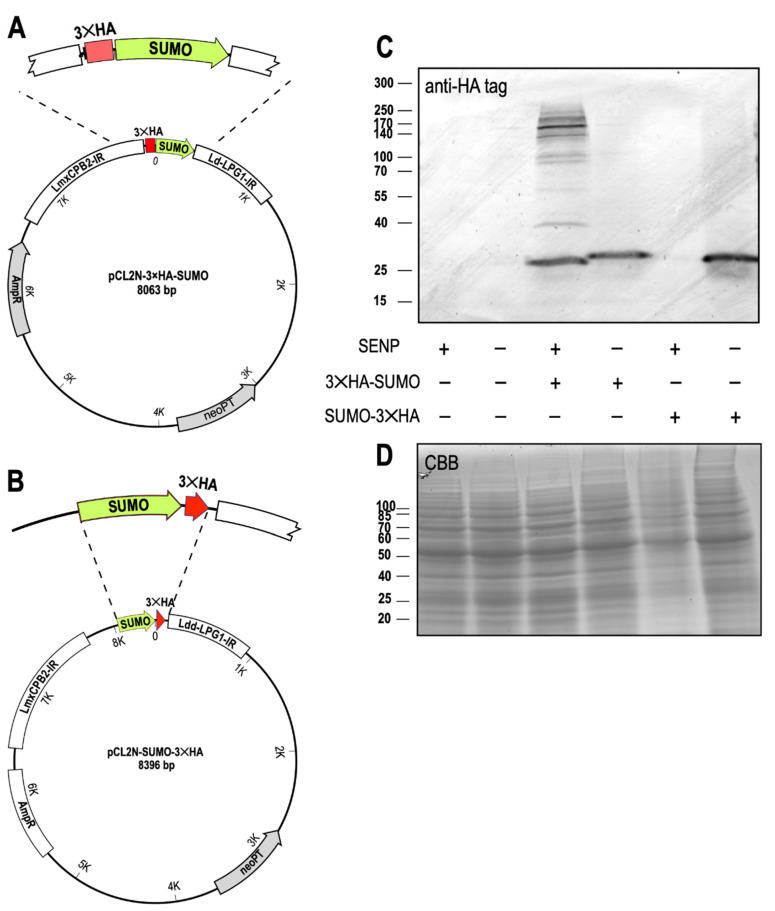
SUMO processing by SENP. (**A**) Schematic drawing of pCL2N-3×HA-SUMO, a plasmid for ectopic expression of SUMO with an N-terminal triple HA tag. (**B**) Schematic drawing of pCL2N-SUMO-3×HA, a plasmid for ectopic expression of SUMO with a C-terminal triple HA tag. (**C**) Western blot of *L. donovani* wild type or SENP^−/−^ null mutants, expressing 3×HA-SUMO or SUMO-3×HA, probed with anti-HA tag antibodies. *n* = 2. (**D**) Coomassie brilliant blue (CBB) staining of replicate SDS-PAGE gel, serving as a loading control. The positions and masses of protein size markers are indicated on the left. Original Western blot and gel images can be seen in Figure S1.

In the next step, we verified that SENP is indeed required for C-terminal processing of SUMO. We constructed plasmids for ectopic expression of SUMO either with an N-terminal 3×HA tag (Figure 4A) or with a C-terminal 3×HA tag (Figure 4B) and transfected them into *L. donovani* wild type and *L. donovani* SENP^−/−^ cl.1. The cells were grown to mid-logarithmic density, collected by centrifugation and lysed in SDS sample buffer. Samples representing equal cell numbers of *L. donovani*, *L. donovani* SENP^−/−^, *L. donovani* (3×HA-SUMO), *L. donovani* SENP^−/−^ (3×HA-SUMO), *L. donovani* (SUMO-3×HA) and *L. donovani* SENP^−/−^ (SUMO-3×HA) were separated by SDS-PAGE, blotted and stained with an anti-HA antibody (Figure 4C). No unspecific HA tag staining was observed for wild type and the SENP^−/−^ mutant. Ectopic expression of 3×HA-SUMO in the wild type background resulted in a band corresponding to 25 kD, not the expected 16 kD of the triple-HA-tagged SUMO. The aberrant migration of SUMO in SDS-PAGE has been described before [43] and explains the observed band. We also observe numerous bands of higher molecular mass. Their spacing and varying intensities does not reflect the incremental size increases expected of SUMO homoconjugates, but rather suggests HA-tagged, SUMOylated substrate proteins. In the SENP^−/−^ background, expression of the same transgene resulted in a slightly larger band, presumably representing the monomeric, non-processed 3×HA-SUMO. No larger HA-tagged bands were detectable, indicating that unprocessed SUMO is incapable of being conjugated to itself or to target proteins.

No HA-tagged proteins are visible when the C-terminally tagged SUMO-3×HA is expressed in the wild type background. Expression of the same chimera in SENP^−/−^ cells, by contrast, yields HA-tagged SUMO. This demonstrates that C-terminal processing of SUMO depends on SENP. We conclude that SENP is required for processing and conjugation of SUMO to itself and/or to other proteins, and establishes C-terminal cleavage of SUMO as a critical step for SUMOylation in *Leishmania*.

### 3.5. SENP-Dependent Processing Determines SUMO Localization

**Figure 5:**
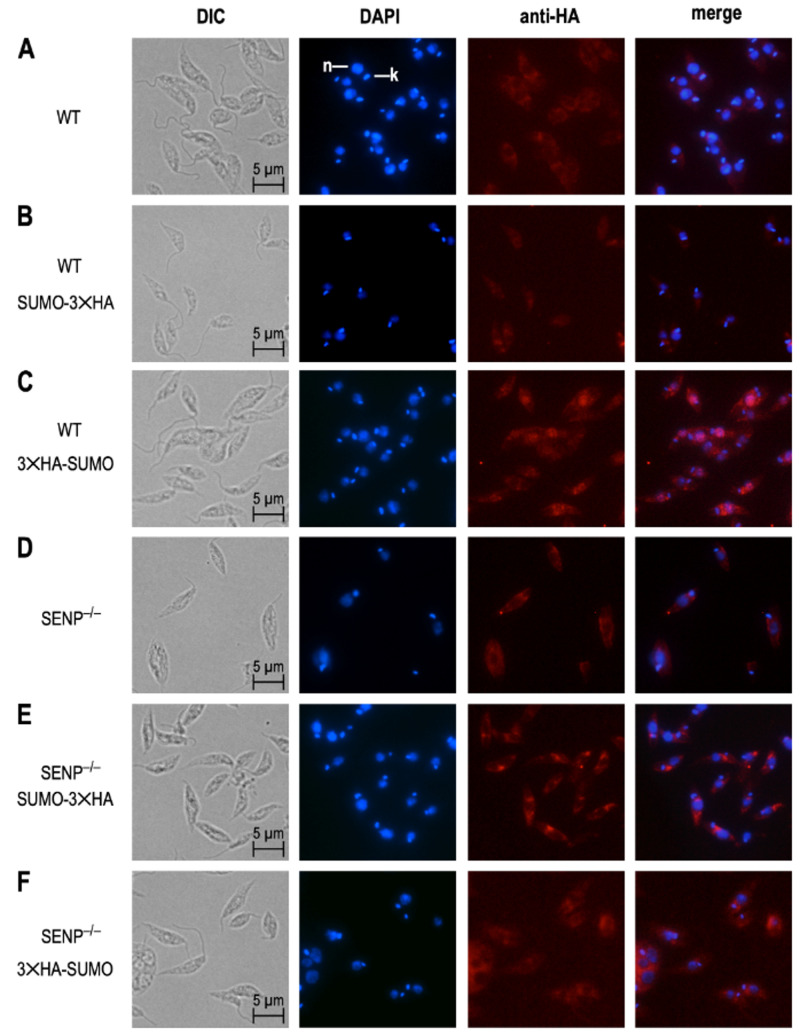
Subcellular localisation of HA-tagged SUMO. Wild type (WT) (**A**) or *L. donovani* SENP^−/−^ (**D**) cells expressing SUMO-3×HA (**B**,**D**) or 3×HA-SUMO (**C**,**F**). Cells were visualized by differential interference contrast (DIC), DAPI staining of nucleus and kinetoplast, and mouse anti-HA antibody/anti-mouse AlexaFluor 594. DAPI and anti-HA images were merged with 50% transparence. Size markers (5 µm) are shown in the DIC images; nucleus (n) and kinetoplast (k) are pointed out in the top DAPI image.

We next investigated the impact of SENP-dependent processing on the subcellular localization of SUMO. For this, promastigotes of six strains, *L. donovani* wild type, *L. donovani* SENP^−/−^, *L. donovani* (3×HA-SUMO), *L. donovani* SENP^−/−^ (3×HA-SUMO), *L. donovani* (SUMO-3×HA) and *L. donovani* SENP^−/−^ (SUMO-3×HA), from logarithmic culture, were spread on glass slides, fixed and stained with DAPI and with anti-HA tag antibody/anti-mouse AlexaFluor 594, followed by immune fluorescence microscopy. As expected, *L. donovani* wild type and *L. donovani* SENP^−/−^ showed no 3×HA-specific staining (Figure 5A,D). We also did not observe a 3×HA-specific signal in *L. donovani* (SUMO-3×HA; Figure 5B), likely due to the cleavage of the C-terminal 3×HA tag in wild type cells. *L. donovani* (3×HA-SUMO) cells showed overlapping staining by DAPI and anti-HA tag antibody, indicating a nuclear localization of 3×HA-SUMO in the wild type.

*L. donovani* SENP^−/−^ (SUMO-3×HA) and *L. donovani* SENP^−/−^ (3×HA-SUMO) both showed cytoplasmic staining. We conclude from this that (i) SENP^−/−^ mutants cannot cleave off the C-terminal 3×HA tag (Figure 5E) and (ii) SENP-mediated cleavage of the C terminus is essential for nuclear localization of 3×HA-SUMO. The lack of SENP therefore prevents C-terminal processing of SUMO, preventing SUMO from attaining or maintaining a nuclear localization.

### 3.6. Growth Phenotypes of SENP Null Mutants

**Figure 6:**
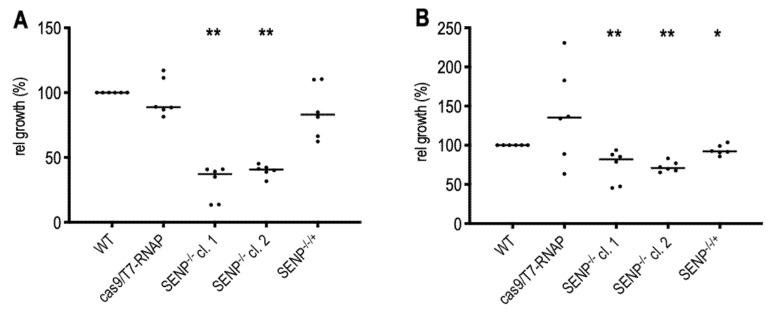
In vitro growth of wild type and mutant *L. donovani*. Cells were seeded at 5 × 10^5^/mL and grown either at 25 °C/pH 7.0 (**A**) or at 37 °C/pH 7.0 (**B**) for 72 h. Final cell densities were normalized against wild type growth (100%). Bars show the median cell growth. *n* = 6 (3 biol. repeats, 2 techn. repeats each). ** = *p* < 0.01; * = *p* < 0.05 (U-test, two-sided).

Given its critical function in SUMOylation, we tested the impact of SENP on the growth of *L. donovani* at different temperatures. *L. donovani*, *L. donovani* (Cas9/T7-RNAP), SENP^−/−^ clones 1 and 2 and the SENP^−/−/+^ add-back strain were seeded at low density, and growth was then monitored over 72 h. Cell densities at 72 h were normalized, with wild type *L. donovani* set at 100% growth. At optimal growth conditions, 25 °C and pH 7.0, both SENP^−/−^ null mutants showed a 50% reduced proliferation compared with wild type and the Cas9-expressing strain. This growth phenotype was reversed by ectopic SENP expression (Figure 6A). At 37 °C, we recorded less, but still significant growth reduction due to the loss of SENP (Figure 6B). This may indicate that SENP function and/or SUMO conjugation is more important at the lower temperature associated with the insect stage.

We also tested the intracellular survival of SENP null mutants in mouse bone marrow-derived macrophages and found no differences in parasite loads compared with wild type parasites (A.B. and C.B., unpublished observations), consistent with a primary role for SENP in the promastigote stage.

## 4. Discussion

As a vector-transmitted parasite, *Leishmania* must adapt to vastly different environments, carbon sources, and antimicrobial defense mechanisms. This must be achieved without differentially regulated RNA synthesis [3,44,45]. Instead, *Leishmania* relies on modulated RNA stability [46], RNA processing [47] and inducible translation [2,5,48] as means of short-term gene expression control. Long-term adaption to changing environments, by contrast appears to be mediated by gene copy number variations, either by chromosomal aneuploidy [6,7] or by amplification of genes and gene clusters [36,49,50]. A third level of gene expression control are PTMs of proteins that may activate or inhibit activities or influence localization. Examples of PTMs are protein kinase mediated phosphorylation of threonine and serine side chains [8,11,13]. Side chain-specific modifications can impact on protein folding or protein–protein interactions. The covalent attachment of modifying polypeptides is another, as yet little understood mode of expression control in *Leishmania*. So far, only the impact of a ubiquitin fold modifier (UFM1) protein was demonstrated [17,51] in *L. donovani*. A similar modifier, small ubiquitin-like modifier (SUMO) was identified and characterized in *Trypanosoma* spp. where it is involved in surface antigen expression and nuclear organization [18,19,52]. Here we describe the *Leishmania* SUMO and SENP orthologs and characterize them by reverse genetic, biochemical and cell biological means.

To the best of our knowledge, SUMO is an essential gene in *L. donovani* promastigotes. Attempts to produce SUMO^−/−^ null mutants by CRISPR-mediated gene editing failed while the same gene replacement tools were successfully employed in a strain carrying ectopic SUMO copies (Figure 2D,E), indicating that null mutants are either non-viable or non-proliferative as promastigotes *in vitro*. It was shown for higher eukaryotes that the SUMO pathways are essential during differentiation processes [53,54], but our literature search did not turn up reports of an outright SUMO gene replacement. This is probably also due to the presence of multiple SUMO genes in mammalian cells [55], which may confound reverse genetics approaches.

Unlike SUMO, SENP appears to be non-essential, albeit with a significant impact on promastigote proliferation at optimal growth temperature, with a smaller effect at mammalian tissue temperatures. Fittingly, the survival of amastigotes within mouse macrophages is unaffected by the loss of SENP. This may indicate an important role of SENP and its clients during logarithmic growth of *Leishmania* promastigotes, but less impact during the slow growth of intracellular amastigotes. Yet, with SUMO C-terminal processing abrogated by the loss of SENP (Figure 4C) and its nuclear localization severely reduced (Figure 5), it surprises that the effect of SENP loss is not equally deleterious as the loss of SUMO. Strong signals for C-terminally tagged SUMO in SENP null mutants (Figure 4C and Figure 5) argue against a SUMO processing pathway using alternative proteases. One must therefore assume, that apart from its role as a conjugated protein modifier, SUMO must have additional, essential functions in *Leishmania*.

SUMO and its processing protease, SENP, are proteins with constitutive, above-average synthesis rates in *Leishmania*, indicating a need for abundance or a high turnover rate. Indeed, SUMO (LinJ.08.0480) showed little changes of abundance during promastigote-to-amastigote differentiation *in vitro* [21], and SENP (LinJ.26.2070) has a constitutive abundance too [24].

Immune fluorescence microscopy of tagged SUMO protein shows a nuclear, but not kinetoplast localization. This localization fully depends on SENP-mediated C-terminal processing (Figure 5). This result is in keeping with reports that show involvement of SUMO in nuclear organization and chromosome segregation [53]. Preliminary data (A.B.), however, show no impact of a SENP loss on the accessibility of *L. donovani* chromatin to micrococcal nuclease digest. This must be seen, however, in the context of the Trypanosomatida having a divergent chromatin structure and nuclear architecture. While the genomic DNA is assembled into 10 nm fibers of nucleosomes, these protozoa lack further condensation of chromosomes into 30 nm solenoid fibers [56]. The function of SUMO in the nucleus may therefore be diverged.

The affinity of HA-tagged SUMO for the nucleus is also a promising possibility to identify SUMOylated proteins from the cytoplasm and the nucleus via immune precipitation of SUMO-target conjugates and subsequent mass spectrometric analysis.

## 5. Conclusions

*Leishmania* parasites express proteins belonging to the SUMO protein modification pathway. The gene coding for SUMO is essential for growth and/or viability of *L. donovani* promastigotes, while the SENP processing enzyme is required for the C-terminal processing of SUMO and its nuclear localization, but dispensable for *L. donovani* viability. The SENP^−/−^ null mutants show a 60% reduced growth at ambient temperature, but less impact at mammalian tissue temperature. No decrease of viability during *in vitro* infection can be observed, indicating a primary role for SENP-dependent SUMOylation in the fast growing promastigote stage. Additionally, the viability of SENP^−/−^ null mutants hints at a vital importance of as yet unknown, SENP-independent functions of SUMO.

## Supplementary Materials

The following are available online at https://www.mdpi.com/2073-4425/11/10/1198/s1, Table S1: Oligonucleotides used for targeting constructs; Table S2. Primers used for RT-qPCR and PCR; and Figure S1: Images of original Western blot and Coomassie Brilliant Blue-stained PA gel.
