# Supplementary material for: The Leishmania donovani SENP Protease Is Required for SUMO Processing but Not for Viability
Source: Genes (Basel). 2020 Oct 14;11(10):1198. doi: 10.3390/genes11101198 (PMC7602377; doi:10.3390/genes11101198)
Supplement: Supplementary file 1 [file genes-11-01198-s001.pdf]

The *Leishmania donovani* SENP Protease is Required for SUMO Processing but not for Viability

Annika Bea[#], Constanze Kröber-Boncardo[#], Manpreet Sandhu[§], Christine Brinker and Joachim Clos*

Bernhard Nocht Institute for Tropical Medicine, Hamburg, Germany
[#]) A.B. and C.K.-B. contributed equally to this work
[§]) current affiliation: Boehringer Ingelheim RCV, Vienna, Austria
*Correspondence to: email: clos@bnitm.de; Tel: +49-40-42818-481

Table S1: Oligonucleotides used for targeting constructs

Name	Sequence (5′-3′)	Application
G00	AAAAGCACCGACTCGGTGCCACTTTTTCAAGTTGATAACGGACTAGCCTTATTTTAACTTGCTATTTCTAGCTCTAAAAC	Generation of SUMO-/- line using CRISPR/ Cas9
LdBPK_080480 downstream reverse	GCTGCGCGTGCGCCACGATGGACGTTGCGACCAATTTGAGAGACCTGTGC	
LdBPK_080480 upstream forward	CCCCTCCCCTCCCTGCCGTCGCGTCGCACGGTATAATGCAGACCTGCTGC	
LdBPK_080480 3′gRNA	GAAATTAATACGACTCACTATAGGGGGAAGGAGGGAGAGTTTGGGTTTTAGAGCTAGAAATAGC	
LdBPK_080480 5′sgRNA	GAAATTAATACGACTCACTATAGGTAGCGAAGAAGCACAGCAAGGTTTTAGAGCTAGAAATAGC	
LdBPK_262070 upstream forward	ATACACACACACACACACGCAAATATACCCgtataatgcagacctgctgc	Generation of SENP-/- line using CRISPR/ Cas9
LdBPK_262070 downstream reverse	CGGTACAGAGCGTTCATGTGCCTCTCTCTCccaatttgagagacctgtgc	
LdBPK_262070 5′sgRNA	gaaattaatacgactcactataggAGAGGGATTCTCCTCCTTTGgttttagagctagaaatagc	
LdBPK_262070 3′sgRNA	gaaattaatacgactcactataggTCTGTCTGCACGAATGCATGgttttagagctagaaatagc	
LdBPK_080480_BamHI_rev	GGAGGGATCCTCATAGCTGCCTCACCACGC	N-terminal tagging of SUMO
LdBPK_080480_NdeI_fwd	GGAGCATATGATGGAGCATCCGGAGCACAC	
KpnI_LdBPK_080480_rev	GGAGGGTACCTAGCTGCCTCACCACGCACC	C-terminal tagging of SUMO
KpnI_LdBPK_080480_fwd	GGAGGGTACCATGGAGCATCCGGAGCACAC	
KpnI_LdBPK_262070_fwd	GGAGGGTACCATGAACTCGCAGCCGTCGAC	pCL2N-SENP
BamHI_LdBPK_262070 rev	GGAGGGATCCTCAGACGCCGCTGGACGACG	pCL2N-SENP
pIR-P-fwd2	GGCTCTGCGTTTCACTTGC	Sequencing primer
pIR-P-rev	GCGAACTGGTCGTAGAAATC	

Table S2. Primers used for RT-qPCR and PCR.

Name	Sequence (5′ to 3′)	Application
5′-UTR-LdBPK_080480.1_EcoRI	GAGGGAATTCATTTAAATAAACCATGTTATCGCGTATG	Genotyping of expected SUMO$^{-/-}$ mutants
LdBPK_080480.1_BamHI	GAGGGGATCCTCATAGCTGCCTCACCACG	
3′-UTR-LdBPK_080480_HindIII	GAGGAAGCTTATTTAAATCAGGCTGCTTGGCTTCCCAC	
LdBPK_080480_NdeI	GAGGCATATGGAGCATCCGGAGCACAC	
LdBPK_262070 qPCR fwd	TATCACCTCTCGATTCCTTGCAG	SENP cDNA detection
LdBPK_262070 qPCR rev	ACAGCTCCTCTTGATGTCGTATC	
Cas9 qPCR fwd	CGAGAAGCTGTACCTGTACTACC	Cas9 cDNA detection
Cas9 qPCR rev	CACGATATGGTCCACATCGTAGT	

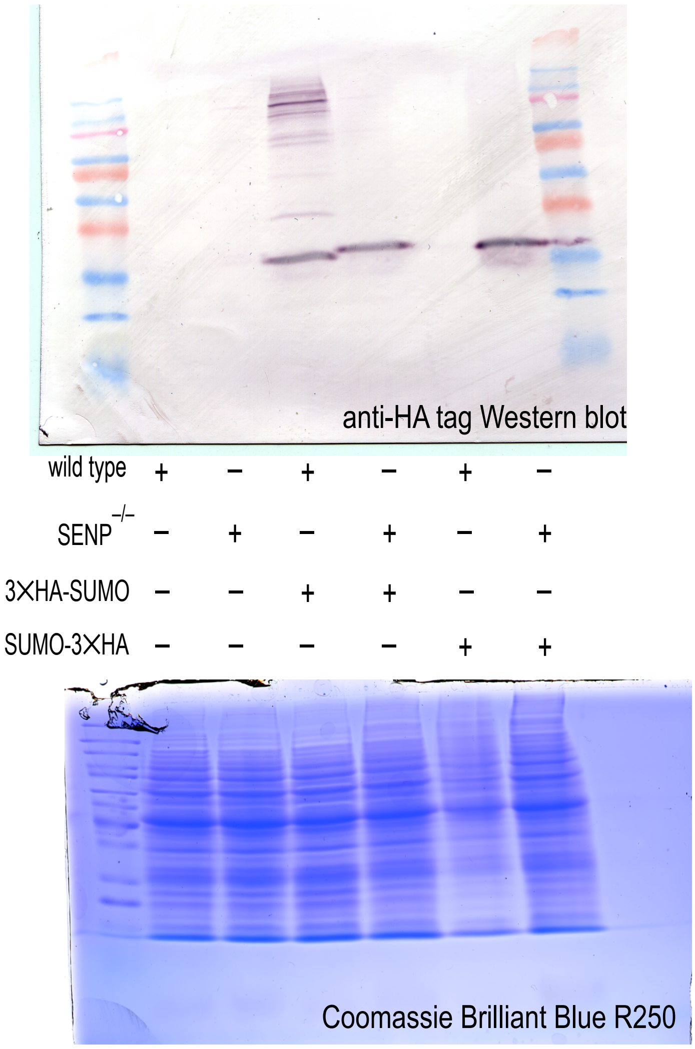

Figure S1: Images of original Western blot and Coomassie Brilliant Blue-stained PA gel.
